# Mapping and manipulating the *Mycobacterium tuberculosis* transcriptome using a transcription factor overexpression-derived regulatory network

**DOI:** 10.1186/s13059-014-0502-3

**Published:** 2014-11-03

**Authors:** Tige R Rustad, Kyle J Minch, Shuyi Ma, Jessica K Winkler, Samuel Hobbs, Mark Hickey, William Brabant, Serdar Turkarslan, Nathan D Price, Nitin S Baliga, David R Sherman

**Affiliations:** Seattle Biomedical Research Institute, 307 Westlake, Seattle, WA 98109-5219 USA; Institute for Systems Biology, Seattle, WA 98109 USA; Interdisciplinary Program in Pathobiology, University of Washington, Seattle, WA 98195 USA; Department of Chemical Engineering, University of Illinois, Urbana, IL USA

## Abstract

**Background:**

*Mycobacterium tuberculosis* senses and responds to the shifting and hostile landscape of the host. To characterize the underlying intertwined gene regulatory network governed by approximately 200 transcription factors of *M. tuberculosis*, we have assayed the global transcriptional consequences of overexpressing each transcription factor from an inducible promoter.

**Results:**

We cloned and overexpressed 206 transcription factors in *M. tuberculosis* to identify the regulatory signature of each. We identified 9,335 regulatory consequences of overexpressing each of 183 transcription factors, providing evidence of regulation for 70% of the *M. tuberculosis* genome. These transcriptional signatures agree well with previously described *M. tuberculosis* regulons. The number of genes differentially regulated by transcription factor overexpression varied from hundreds of genes to none, with the majority of expression changes repressing basal transcription. Exploring the global transcriptional maps of transcription factor overexpressing (TFOE) strains, we predicted and validated the phenotype of a regulator that reduces susceptibility to a first line anti-tubercular drug, isoniazid. We also combined the TFOE data with an existing model of *M. tuberculosis* metabolism to predict the growth rates of individual TFOE strains with high fidelity.

**Conclusion:**

This work has led to a systems-level framework describing the transcriptome of a devastating bacterial pathogen, characterized the transcriptional influence of nearly all individual transcription factors in *M. tuberculosis*, and demonstrated the utility of this resource. These results will stimulate additional systems-level and hypothesis-driven efforts to understand *M. tuberculosis* adaptations that promote disease.

**Electronic supplementary material:**

The online version of this article (doi:10.1186/s13059-014-0502-3) contains supplementary material, which is available to authorized users.

## Background

*Mycobacterium tuberculosis* (MTB) is a remarkably successful human pathogen, with a global burden of over 1.5 billion latently infected individuals and 1.3 million deaths due to tuberculosis (TB) per year [1]. To survive within the hostile environment of the human host, MTB must sense and respond to a wide variety of microenvironments including naïve and activated macrophages, dendritic cells, and evolving conditions within different types of granulomas [2]. Regulation of these responses begins by controlling the expression of transcripts that combine to form transient, often overlapping networks and collectively coordinate adaptation to shifting host-mediated stresses. MTB employs a set of approximately 200 transcription factors (TFs) and DNA binding proteins to mediate signals from the changing environment and, along with the RNA degradation machinery [3], dictate the expression profiles of genes. Some MTB TFs have been characterized previously by a variety of approaches including gene knockout, overexpression, chromatin immunoprecipitation, and an assortment of *in silico* approaches [4-18]. The majority, however, have not been studied and have unknown regulatory targets and biological roles.

To investigate the MTB transcriptional landscape in a systematic manner, we developed a high-throughput approach to identify the genes controlled by nearly all predicted MTB TFs. We individually cloned and conditionally overexpressed 206 MTB TFs to induce the regulatory signature of each one. This signature includes both genes directly controlled by proximal binding of the TF as well as genes indirectly influenced via a cascade of interactions triggered by the TF. Using this approach we identified the sets of genes affected by TF overexpression (TFOE) and assembled them into an easily searchable map of transcriptional regulation in MTB. This network defines the influence of 183 TFs and complements a comprehensive TF-DNA binding network and transcriptional modeling efforts performed in parallel [19,20]. Our data agree well with the small set of MTB regulons previously reported in the literature, indicating that overexpression of TFs can stimulate native gene expression even in the absence of co-stimulatory factors and validating our overall approach. We show that the number of regulated genes per TF varies by nearly 1,000-fold, and that the majority of expression changes act to repress basal transcription. We find evidence of regulation for 70% of all MTB genes, two-thirds of which are controlled by more than one TF. Identities of regulated genes and their associated gene ontology categories suggest functional roles for many TFs and their regulons. We then assessed the fidelity of network-derived predictions, rewiring the MTB transcriptome selectively to confer inducible phenotypic drug resistance and testing growth rate predictions of individual TFOE strains. Altogether, this work offers systems-level insight into the transcriptome of a devastating bacterial pathogen, delineates the functional impact of numerous individual TFs, and should stimulate additional efforts to understand MTB adaptations that promote disease.

## Results

### Construction and expression profiling of a library of TFOE strains

**Figure 1:**
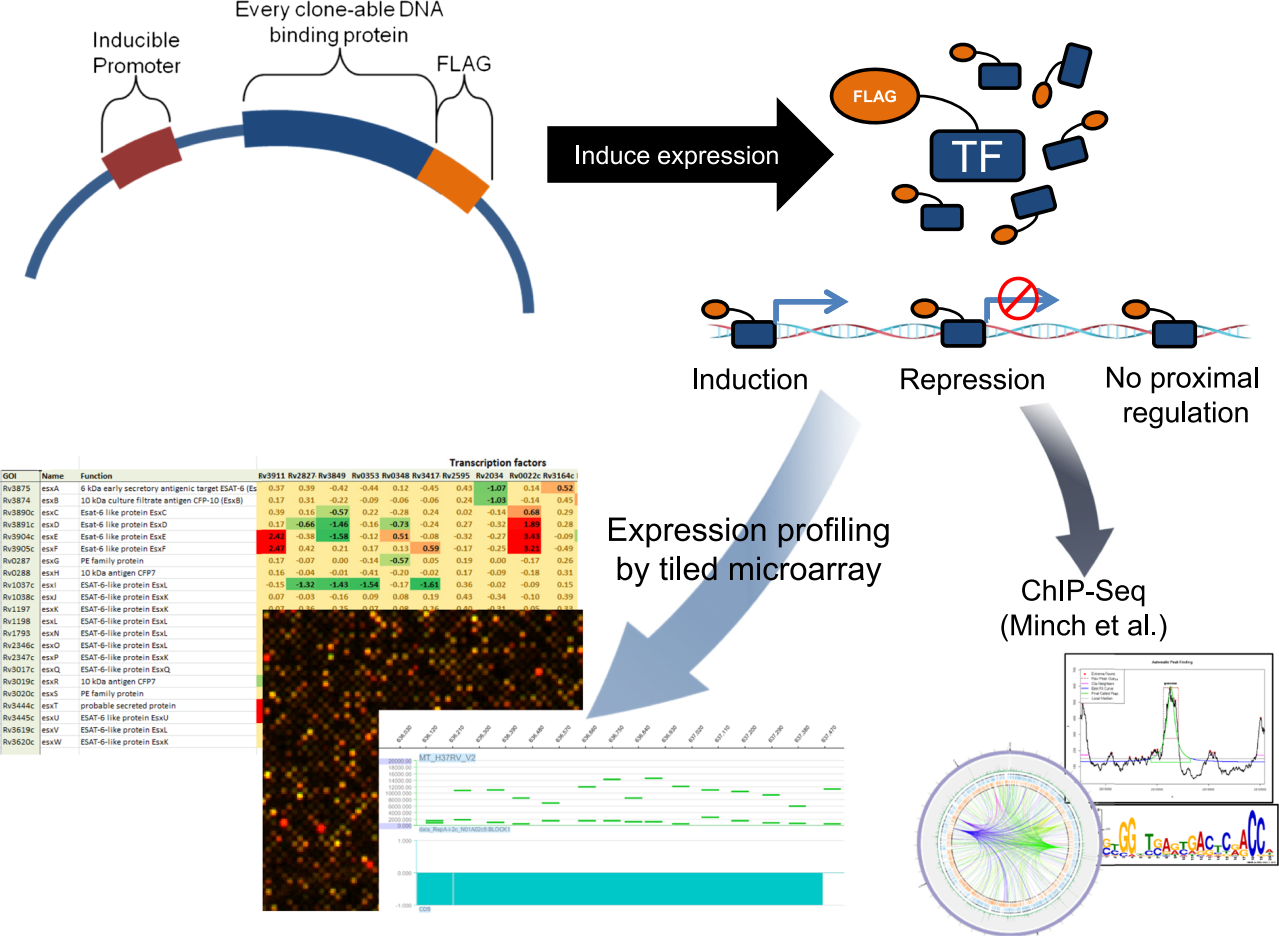
**Schematic diagram of a high-throughput screen of transcription factor overexpression constructs.** We cloned 206 of 214 annotated DNA binding proteins (TFs) into a plasmid that placed the tagged protein under control of a tetracycline inducible promoter and fused the TF to a FLAG tag. Each of these TFs was then induced for one doubling period (approximately 18 h) and analyzed via expression profiling and ChIPseq [19]. Expression profiles were characterized using microarrays that covered both strands of the genome with a probe every approximately 100 bp.

All known and predicted TFs were selected for cloning based on previously characterized function, sequence similarity to known TFs in other organisms, and protein domains with DNA binding motifs (Figure 1, Additional file 1: Table S1). Tuberculist [21] annotated 178 genes as TFs and an additional 13 as sigma factors. We excluded a methyltransferase (Rv0560c) and three putative MoxR orthologs (Rv1479, Rv3164c, and Rv3692), as those genes do not have DNA binding domains and appear to be mis-annotated. We then supplemented this list of 187 with 27 additional genes that matched to transcriptional regulation-relevant COG domains [22]. Of the set of 214 candidates, 206 were subcloned into a vector under the control of an anhydrotetracycline (ATc) inducible promoter to allow overexpression of each TF independent of the native stimulatory factors unique to each TF. The resulting set of TFOE plasmids was transformed into the MTB strain H37Rv. The remaining eight TF genes have resisted our efforts at cloning thus far.

Overexpression assays were performed under standardized culture conditions (see Methods and [23]) in order to facilitate transcriptome-wide comparisons and potentially to identify activating environmental conditions and/or small molecule triggers of these TFs. TF overexpression was induced for a duration time of approximately one cell doubling (18 h) with 100 ng/mL of anhydrotetracycline (ATc) and cells were subsequently harvested for transcriptome analysis and ChIP-seq, as described separately [19]. Global transcriptional changes were assayed using densely tiled microarrays with 60mer probes for both strands of the genome at an average density of one probe per 100 nucleotides. This resulted in a compendium of 702 transcriptome profiles for 206 strains, representing a sum total of 95 million data points that we incorporated into a transcriptional regulatory network of MTB.

### TFOE defined regulatory effects

**Figure 2:**
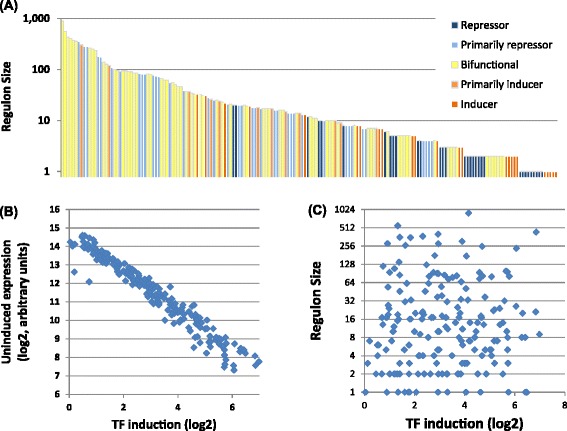
**Features of the TFOE dataset. (A)** TFOE-induced transcriptional changes vary widely in size and composition. Each of the 183 TFOE regulons (genes differentially expressed (DE) two-fold with a FDR adjusted *P* value <0.01) is represented as a single bar indicating the total number of genes DE. Each column representing a TF was further characterized as either entirely or primarily an inducer (red), repressor (blue), or bifunctional regulator (yellow). **(B)** Ectopic induction inversely correlates with baseline expression level. The level of induction for each TF is strongly correlated with the uninduced expression level, however neither of those variables is correlated with the size of the regulon **(C)**.

Altogether we identified 9,335 instances where TF overexpression led to a significant gene expression change (two-fold change, *P* value ≤0.01), driven by 183 of the 206 TFs assayed (Figure 2A, Additional file 1: Table S2). Each TFOE regulon includes both direct interaction at promoter regions and indirect effects, providing a holistic picture of a TFs role in a system-wide context. We expected that some TFs would be inactive in the absence of their physiological trigger, but only approximately 10% of TFOE strains (23 of 206) failed to yield any genes with significantly altered expression.

The level of induction for each TF is strongly influenced by the baseline expression of that gene (Figure 2B). TFs that are highly expressed prior to induction were not induced much further, whereas TFs expressed at low levels were induced up to 100-fold. In nearly all cases, after induction the TF was among the more abundant transcripts in the cell. However there were on average 40 genes more highly expressed in each case, suggesting that TF overexpression did not result in artificial saturation of the microarray.

To assess if the inducible promoter and standard growth condition that we employed could in some cases result in TF overexpression that exceeds physiological levels, we assembled a collection of 2,483 publicly-available MTB gene expression profiles [20] and compared the level of induction seen in the TFOE experiments with the largest fold change of the relevant TF in any previously published condition. For 82% of TFs there was at least one condition where the level of induction was equal to or larger than we report here, and for 94% of TFs the level of induction was no more than 2X higher than the largest previously reported change (Additional file 2: Figure S1).

### Characteristics of the TFOE dataset

**Figure 3:**
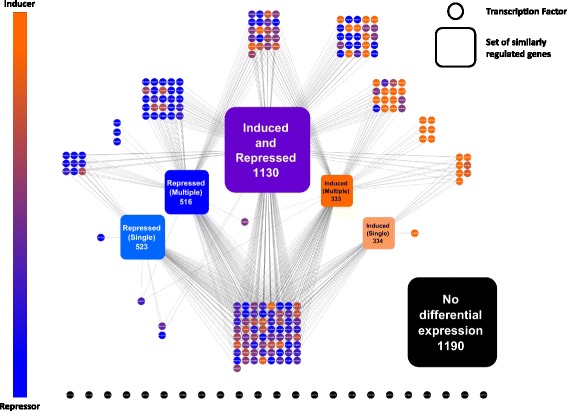
**Manually constructed TFOE network.** Genes were grouped into sets with similar regulation patterns and the interaction of each TF with each set was mapped. The size of each set of genes is indicated beneath the gene set name. The color of each TF indicates whether the regulatory influence of that is primarily to repress (blue) or induce (orange) genes. Genes repressed by multiple TFs and those with no change in expression were enriched for essential genes, many of which have GO terms assigned to them.

When examined in aggregate, some features of the TFOE regulons stand out. The number of regulated genes varied over nearly three orders of magnitude (Figure 2A, bar height). Overexpression of one TF, Rv0023, induces 488 genes and represses 404, leading to differential expression of nearly a quarter of the genome. At the other extreme, 17 TFs changed the expression of only a single gene and for 23 we could identify no regulated genes. Four of the TFs with only a single responsive gene are induced to a larger fold change than Rv0023, highlighting the general phenomena that the number of genes differentially regulated did not correlate with the level of induction (Figure 2C) or with uninduced expression levels (data not shown). These results also suggest that overexpression of these TFs does not induce a common stress response. Most of the TFs were bifunctional, with some downstream genes induced and others repressed. TFs acting exclusively or primarily as repressors are nearly twice as common as inducers (Figure 2B, bar color, Figure 3). Correspondingly, 57% of all instances of altered expression were repressions, consistent with the pattern of regulation seen in the well-studied model bacterium, *E. coli* [24].

### Comparison of TFOE results with existing datasets

To assess the fidelity of our results, we compared 12 previously defined MTB putative regulons, with the TFOE-derived regulatory influences of these TFs. Ectopic induction necessarily masks potential autoregulation, as auto-induction of the native gene is difficult to distinguish from induced expression from the plasmid, so the TF-encoding gene was excluded from the comparisons. The majority of TFOE-defined regulons overlap significantly with those previously identified (Table 1). For example, overexpressing DosR in aerobic conditions produces induction of nearly every gene previously included in the DosR regulon (45 of 48 genes) [25], which was defined using a DosR deletion mutant and hypoxic stress [15,26]. Additionally, two previously characterized regulons of cholesterol metabolism, KstR and KstR2, overlap very significantly with their TFOE derived regulons (*P* value <0.001). On average, the genes triggered by TF overexpression included 70% of genes in previously characterized regulons (*P* value on average less than 0.001). In two-thirds of cases, the number of genes regulated by TFOE is substantially larger than the corresponding regulons described in the literature. Perhaps by inducing TFs we were better able to capture secondary/indirect regulation when compared to gene knockout or *in silico* studies. Only two previously reported regulons, from Rv0195 and Rv2034, showed poor overlap with the TFOE dataset. Both are associated with the MTB Enduring Hypoxic Response (her) [27] and might therefore require reduced oxygen tension as a signal to trigger their activity.

**Table 1:** **Regulons culled from the literature compared to TFOE defined regulons**

**TF**	**Name**	**Type of analysis** ^**a**^	**Reference regulon** ^**b**^	**TFOE regulon** ^**c**^	**Overlap** ^**d**^	***P*** **value**	**Reference**
**Rv0022c**	*whiB5*	OE	61	436	59	<0.001	[4]
**Rv0182c**	*sigG*	OE	7	20	4	<0.001	[5]
**Rv0195**		KO	179	27	1	0.708	[6]
**Rv0465c**	*ramB*	KO	2	15	1	0.007	[8]
**Rv0586**	*mce2R*	KO	4	11	4	<0.001	[9]
**Rv1909c**	*furA*	KO	1	12	1	0.003	[11]
**Rv2034**		Various	11	67	0	1.000	[12]
**Rv2359**	*furB*	KO	23	6	5	<0.001	[13]
**Rv3124**	*moaR*	OE	4	9	4	<0.001	[14]
**Rv3133c**	*dosR*	KO	48	127	45	<0.001	[15]
**Rv3557c**	*kstR2*	KO	15	19	15	<0.001	[16]
**Rv3574**	*kstR*	KO	70	74	32	<0.001	[16]

For each transcription factor set of genes differentially expressed by overexpression was compared to a previously reported regulon.

^a^The type of analysis done in the reference (OE, KO, or various).

^b^The number of genes in the previously published regulon.

^c^The number of genes differentially expressed in our assay.

^d^The number of genes that change in both our assay in prior reports.

KO, knockout; OE, overexpression.

### Network model of the MTB transcriptional network

Using Cytoscape [28], we manually constructed a network of TFs and targets that reveals a highly interconnected landscape with a complex pattern of regulatory influences (Figure 3). This network divides the MTB genome into six sets of similarly regulated genes: genes that are exclusively induced or repressed; those both induced and repressed; and those with no change in expression in response to overexpression of any TF. Genes that are only repressed or induced can be further separated into those regulated by a single TF as opposed to multiple TFs. We then showed the interaction, if any, with each of those gene sets for every TF assayed. Of the 4,026 genes in MTB, the majority (70%) change expression in response to overexpression of at least one TF, and two-thirds of those are regulated by more than one TF.

To understand better the underlying differences in the sets of genes with similar patterns of regulation we looked for gene ontology (GO) terms that were enriched in each set using the R application TopGO (Additional file 1: Table S3). The 636 solely induced genes were not enriched for any GO terms, suggesting that their functional distribution matches that of the MTB genome as a whole. Exclusively repressed genes were broadly enriched in GO terms associated with growth and metabolism. In particular, those genes regulated by multiple repressors are enriched in terms involved in energy production through central metabolism. Genes with more complex regulation (–that is, those that were induced in response to some TF overexpression and repressed in response to others) were enriched for four GO terms, all linked to synthesis and use of acyl carrier proteins.

In contrast, genes that did not change expression in any of the TFOE experiments had 272 GO terms enriched - 10 times as many as the other categories combined. These terms include many unrelated categories, including the essential processes of DNA synthesis and repair, protein synthesis, and ATP synthesis. We therefore assessed the behavior of essential genes [29] in the TFOE dataset. We found that the more often a gene’s expression was regulated the less likely it was to be essential. In fact, genes with no changes in expression were 50% more likely to be essential than random (Additional file 1: Table S4).

### Gene ontology terms significantly enriched in TFOE regulons

To assess the potential role of each TF, we performed gene ontology (GO) enrichment analyses on their regulated genes. Very small regulons can appear to be highly enriched if only a single gene falls by chance into an uncommon GO term, so this analysis was limited to the 130 TFs with at least five genes differentially regulated after TF overexpression. For similar reasons, this analysis was limited to GO terms with at least three member genes.

Enrichment of one or more GO terms was evident in 67 of the TFOE regulons (Additional file 1: Table S5). The few previously well-characterized TFs were enriched for expected GO terms. For example, the genes induced by DosR include the Rv0082-87 operon, which leads to an enrichment of terms related to electron transport (GO:0003954); and the ArgR regulon was enriched for small molecule biosynthesis of nitrogen-containing compounds (GO:0006807). In addition, many of the TFs with no previously identified function have putative roles suggested by the enriched GO terms. For instance, the TF Rv1990c is strongly induced by hypoxic stress [27], but has no identified role or regulatory function. We found that the TFOE-identified Rv1990c regulon is enriched for genes linked to DNA damage repair (GO:0042578), DNA synthesis (GO:0006281), and stress response (GO:0006950), suggesting that it may be involved in protecting the organism from DNA damage under hypoxic non-replicative conditions. Similarly, the TF Rv0023 is poorly studied at present. We found that the Rv0023 regulon is enriched for regulation of NAD reductases (GO:0016655). Rv0023 represses the type I NADH dehydrogenase (*nuoD-N*), but induces the alternate enzymes *ndh* and *ndhA*. Interestingly, *ndh* is essential for replenishing NADH during hypoxic stress [30], and the *nuo* operons are repressed in hypoxia [23,27], suggesting that Rv0023 has a heretofore unappreciated role in the MTB adaptation to reduced oxygen tension.

### TFOE network predicts function and phenotype of a regulator of isoniazid susceptibility

**Figure 4:**
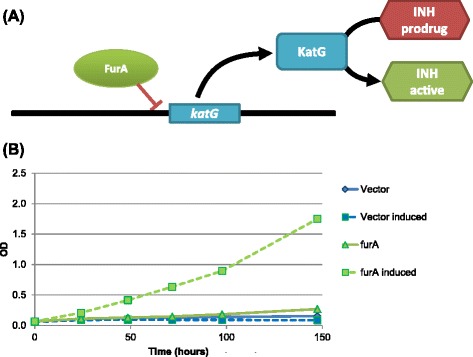
**Isoniazid susceptibility regulator predicted from the TFOE dataset. (A)** KatG converts the prodrug isoniazid (INH) into its active form. One TF, Rv1909c, repressed *katG* when overexpressed, which should lead to reduced levels of KatG, less efficient conversion of INH, and a reduced effect of INH. **(B)** We confirmed this prediction by showing that, in the presence of twice the MIC of isoniazid (0.2 μg/mL), the *furA* TFOE strain was able to grow only when the TF was induced. This increased resistance to isoniazid was not seen in a control strain carrying the parent empty-vector plasmid.

The TFOE regulatory map allows rapid identification of potential regulators of genes and gene sets, and the TFOE strains (available from BEI Resources: NR-46512) can be used to help form and test hypotheses of gene and regulatory function. To demonstrate the potential of these tools we explored the regulation of *katG* (Rv1908c), which encodes the catalase/peroxidase that converts isoniazid prodrug to its active form and is therefore essential for activity of this front-line TB drug [31]. Querying the TFOE dataset revealed that the repressor *furA* (Rv1909c) is the only transcriptional regulator of *katG*. These genes lie in an operon along with Rv1907c. Autoregulation of this operon by FurA has been suggested in MTB [32] and demonstrated in *M. smegmatis* using a deletion of the orthologous gene [11]. We found that over-expression of *furA* had limited transcriptional impact: repression of three genes other than *katG*, including the next gene downstream (Rv1907c); and induction of seven genes, three of which are in an operon of ribosomal proteins. To test if this transcriptional change resulted in reduced sensitivity to isoniazid, we induced a *furA* overexpressing strain before adding isoniazid. We found that the strain overexpressing *furA* grew in the presence of a concentration of isoniazid that completely inhibited growth of uninduced strains (Figure 4).

### TFOE expression data predict MTB strain growth rates

**Figure 5:**
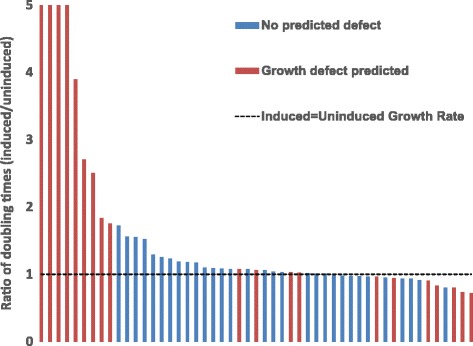
**TFOE expression data mapped onto a a model of MTB metabolism predicts growth restriction.** The gene expression from the TFOE dataset was binarized and applied as constraints on simulations using a MTB genome-scale metabolic model [33]. The growth rates of a set of 51 TFOE strains were measured in the presence and absence of TF overexpression. Each bar shows the ratio of growth rates (uninduced/induced) for a given TF, and the strains predicted to have restricted growth are colored red. Of the 10 strains with the largest increase in doubling time, nine were successfully predicted using this approach.

We mapped transcriptional profiles generated from the TFOE strains onto a published genome-scale metabolic model [33] of MTB to generate condition-specific metabolic models that predict growth rates of the TFOE strains (see Methods for details). To demonstrate the utility of these models, we compared the model-predicted growth phenotypes with experimental growth data for 51 TFOE strains, and we compared the ratio of the uninduced vs. induced growth rates for each strain to the growth ratios predicted by their corresponding TFOE condition-specific metabolic models. Figure 5 shows the measured growth ratios of the TFOE strains, color-coded by whether the corresponding TFOE condition-specific metabolic models predicted a growth defect. The TFOE condition-specific metabolic models demonstrated a statistically significant predictive ability to identify strains with growth ratio of greater than the 85% quantile (corresponding to 1.8-fold reduction), with sensitivity = 1.0 and specificity = 0.72 (*P* <0.001, Fisher’s Exact Test), and TFOE strains with a predicted growth defect had significantly greater uninduced vs. induced growth ratios than strains without a predicted growth defect (*P* = 0.01, t-test). Growth defects were associated somewhat with repression of essential target genes (sensitivity = 0.88, specificity =0.56, *P* = 0.0496, Fisher’s Exact Test; *P* = 0.0498, t-test comparing growth ratios of TFOE strains with repressed targets and those without), but the TFOE condition-specific metabolic models achieved higher performance and improved confidence. Therefore, the TFOE datasets contextualize the metabolic model to gain additional physiological insight and predictive power.

## Discussion

MTB is arguably the world’s most successful bacterial pathogen, adapting readily to changing conditions within the human host and responsible for one death every 25 s [2]. We describe here a transcriptional regulatory network that includes 183 TFs regulating 2,834 genes via 9,335 discrete regulatory events. For comparison, the best characterized prokaryotic regulatory network is arguably that of the model organism *E. coli*, which is catalogued in the actively curated RegulonDB [24] that includes data from over 5,000 publications and identifies 3,122 regulatory interactions from 197 TFs. The number of regulated genes per TF in MTB varies from one to nearly 1,000 and most TFs are bifunctional, producing both increases and decreases of selected genes. Altogether however, 57% of gene expression changes repress transcript levels. We found no correlation between the level of TF expression or its level of induction and the number of attendant gene expression changes (Figure 2).

About 11% of MTB TFs (23 total) produced no transcriptional changes when overexpressed. As mentioned above, this could in some cases result from the absence of a needed activating cofactor. Alternatively, a TF may be present at a saturating level under baseline conditions, in which case the addition of more TF would have no further impact. It is also possible in some cases that the cloned TF was inactivated by interference from the FLAG tag we added or through other artifacts introduced during cloning. However, these issues were likely minor. Both the high percentage of induced TFs that triggered expression changes and the strong overlap with previously reported regulons (Table 1) argue for the general validity of the TFOE approach and results. For the 12 previously studied TFs, we sometimes detected more downstream expression changes than in earlier reports. This is not surprising given that the earlier reports stem from a wide range of experimental conditions and methods of varying sensitivity, which we compare to a single, highly sensitive transcriptomic platform [23,25]. A few TFs may produce exaggerated effects as a consequence of inflated overexpression. However, all but 13 TFs (6% of total) were induced to within two-fold of expression levels previously reported in other experiments (Additional file 2: Figure S1).

The TFOE expression data described here are complemented by ChIP-seq experiments done in parallel to map the DNA-transcription factor binding sites [19]. The TFOE and DNA-binding regulatory networks exhibit significant overlap, with nearly 1,000 cases where TF binding within promoters could be tied directly to significant gene expression changes (*P* value <1 × 10^10^; Additional file 1: Table S2, and [19]). The majority of individual TFs for which we generated both expression and ChIP-seq data show significant overlap (*P* value <0.05) that will likely increase as additional data are collected and incorporated. For example, we hypothesize that the physical TF-DNA binding measured in ChIP-seq may sometimes require additional condition-specific co-factors (sigma factors, small molecules, and so on) not present in our experiments to produce expression changes. In addition, the TFOE expression changes were measured 18 h after TF induction, allowing ample time for indirect transcriptional effects to accumulate.

To visualize the MTB transcriptome, we manually constructed a Cytoscape [28] network portraying the influence of individual TFs on groups of similarly regulated genes. As evident in Figure 3, at least 50% of all MTB genes are subject to multiple transcriptional influences. Genes that were not regulated in TFOE experiments and those controlled by a single repressor were more likely to be essential. Essential genes may be under more complex regulation than is revealed in TFOE experiments, with their expression levels potentially less susceptible than other genes to change within cells.

The TFOE system suggests a new approach to exploring transcriptional regulation and phenotypes in MTB. Instead of perturbing single genes we can now leverage the multiplicative effect of TFs that evolved to rewire the transcriptome in response to complex and shifting signals. TFOE data can be readily searched for regulators of specific genes and gene sets of interest, producing testable hypotheses as with FurA regulation of the isoniazid activator KatG (Figure 4). Similarly, we identified 67 TFs whose regulated genes were enriched in particular functional categories (Additional file 1: Table S5), suggesting further experiments to test regulon function. We can also merge TFOE transcriptional data with other systems-level analyses to generate robust and testable condition-specific phenotypic predictions (Figure 5) [19,20]. The fidelity with which TFOE transcriptional signatures mapped onto the previously described MTB metabolic model [34] predicts growth defects highlights the utility of both the TFOE dataset and metabolic models, as well as the synergy to be realized in combining methods. We are currently employing such approaches to investigate regulatory modules responsible for adaptation to physiologically relevant stresses, both *in vitro* and *in vivo*.

## Conclusion

The TFOE dataset and strain library presented here provide valuable information and novel tools for exploring the transcriptome of MTB, identifying sets of co-regulated genes, and generating/testing hypotheses by simultaneously manipulating co-regulated sets of genes. All tools, reagents, and data described here are available through public repositories. The TFOE strains are available through the BEI strain repository at ATCC ([35], NR-46512). Accessing large datasets like the TFOE expression data can be difficult when the data spread over thousands of genes and hundreds of regulators. To address the difficulties usually associated with accessing large datasets, we have designed a simple Excel spreadsheet for querying TFOE data to find regulators of specific genes or sets of genes. This spreadsheet and all associated data are available in searchable form [36].

## Methods

### Expression vectors and strains

Transcription factor overexpressing strains were generated as described previously [23,25]. In brief, we attempted to clone 214 putative DNA binding genes in the *M. tuberculosis* genome into a tagged, inducible vector using a Gateway Entry Clone library (PFGRC/Colorado State University under NIAID contract HHSN266200400091c, currently available from BEI). For a small set of TFs that were not in the library we created entry clones *de novo*. Eight genes proved recalcitrant to sub-cloning efforts and so were removed from subsequent analyses leaving 206 TFs used in this study. Each of these entry clones was then sub-cloned into a vector via a Gateway cloning recombination cassette (kind gift of Eric Rubin) that placed the TF under control a tetracycline inducible promoter [37] and added a C-terminal FLAG epitope tag. This construct was then transformed into *M. tuberculosis* H37Rv using standard methods. These strains are available from the BEI strain repository at ATCC ([35], NR-46512).

### Culturing conditions

*M. tuberculosis* strain H37Rv was cultured in Middlebrook 7H9 with the ADC supplement (Difco), 0.05% Tween80 at 37°C with constant agitation. Strains containing the ATc-inducible expression vector were grown with the addition of 50 μg/mL hygromycin B to maintain the plasmid. All experiments were performed under aerobic conditions and growth was monitored by OD600. At an OD600 of 0.35, expression of a gene of interest was induced for the approximate duration of one cell doubling (18 h) using an ATc concentration 100 ng/mL culture.

### RNA isolation

RNA was isolated as described previously [27,38]. Briefly, cell pellets in Trizol were transferred to a tube containing Lysing Matrix B (QBiogene, Inc.), and vigorously shaken at max speed for 30 s in a FastPrep 120 homogenizer (Qbiogene) three times, with cooling on ice between steps. This mixture was centrifuged at max speed for 1 min and the supernatant was transferred to a tube containing 300 μL chloroform and Heavy Phase Lock Gel (Eppendorf North America, Inc.), inverted for 2 min, and centrifuged at max speed for 5 min. RNA in the aqueous phase was then precipitated with 300 μL isopropanol and 300 μL high salt solution (0.8 M Na citrate, 1.2 M NaCl). RNA was purified using an RNeasy kit following manufacturer’s recommendations (Qiagen) with one on-column DNase treatment (Qiagen). Total RNA yield was quantified using a Nanodrop (Thermo Scientific).

### Microarray analysis

RNA was converted to Cy dye-labeled cDNA probes as described previously [27]. For all microarrays described here, 3 μg of total RNA was used to generate probes. Sets of fluorescent probes were then hybridized to custom NimbleGen tiling arrays consisting of 135,000 probes spaced at approximately 100 bp intervals around the M. tuberculosis H37Rv genome (NCBI Geo Accession #: GPL14896). These arrays provide 105,000 data points for each expression profile covering approximately 13,000 sense, antisense, and intergenic genome features. For background we compared the expression levels of these probes to a set of 30,000 randomers of equivalent GC distribution. These arrays are no longer commercially available, but arrays with identical probes are available from Agilent (Array ID ‘MTB.tiled.3.2013’). Arrays were scanned and spots were quantified using Genepix 4000B scanner with GenePix 6.0 software. Each TFOE strain was analyzed a minimum of three times. These data were exported to NimbleScan for mask alignment and robust multichip average (RMA) normalization [39]. Subsequent statistical analysis and data visualization were carried out using Arraystar software. To compare against a standard, baseline, expression set, median expression values were calculated for all genes across all 698 input microarrays. Altered gene expression was considered significant if it produced a moderated t-test *P* <0.01 after Benjamini Hochberg multiple testing correction. Array data are available at NCBI-GEO, series GSE59086 and [36].

### Mapping TFOE expression data to metabolism

We generated condition-specific metabolic models based on the transcriptional profiles of TFOE strains and a published genome-scale metabolic model of MTB [21] using the iMAT approach implemented in the COBRA Toolbox [40-42]. The transcriptional profiles of all replicates for each TFOE strains were summarized and binarized such that genes with negative fold change relative to the median over all experiments in at least 75% of the replicates are designated ‘off’, and the remaining genes are designated ‘on’. The binarized transcriptional profiles of each TFOE strain were mapped to the genome-scale metabolic model to generate a predicted growth and reaction flux profile that obeys stoichiometric and thermodynamic constraints and maximizes the number of reactions with nonzero flux activity that map to ‘on’ genes and minimizes the number of reactions with nonzero flux that map to ‘off’ genes. The resulting simulated growth rate of each TFOE condition-specific model was compared to the simulated wild-type growth rate simulated from the genome-scale metabolic model. The TFOE-specific models yielded essentially binary simulated growth rates, with ratios relative to wild-type of either less than 0.01 or greater than 0.95. Therefore, TFOE strains with models that predicted growth rates of less than 95% of wild-type were deemed to predict a growth defect. To assess predictive performance of the models, we set TFOE strains with experimental uninduced vs. induced growth ratios above the threshold value as having a growth defect and those below the threshold of having no growth defect, and we calculated sensitivity as the fraction of strains correctly predicted to have a growth defect $\left(\frac{true\kern0.5em  positive}{true\kern0.5em  positive+ false\kern0.5em  negative}\right)$ and specificity as the fraction of strains correctly predicted not to have a growth defect $\left(\frac{true\kern0.5em  negative}{true\kern0.5em  negative+ false\kern0.5em  positive}\right)$.

## Additional files

Additional file 1: Table S1. Table of transcription factor overexpression strains and characteristics of the resulting transcriptional impact. **Table S2.** Expression changes triggered by transcription factor overexpression. **Table S3.** Gene ontology enrichment in sets of similarly regulated genes. **Table S4.** Level of regulation compared to essentiality. **Table S5.** Gene ontology enrichment in the set of differentially expressed genes for each TF. **Table S6.** Predicted and observed growth rates of TFOE strains. Additional file 2: Figure S1. Comparison of TFOE induction to previously published expression analyses. For each TF the largest fold change was found among the collection of 2,483 expression profiles and that change was compared to the TFOE induction. A histogram of the differences shows a large majority of TFs are not induced beyond what is seen in at least one other condition (the bins under the bracket).

## References

[1] World Health Organization (2013). Global Tuberculosis Control: WHO Report 2013.

[2] Russell DG, Barry CE, Flynn JL (2010). Tuberculosis: what we don’t know can, and does, hurt us. Science.

[3] Rustad TR, Minch K, Winkler J, Brabant W, Reiss D, Baliga N, Sherman DR (2012). Global analysis of mRNA stability in Mycobacterium tuberculosis. Nucleic Acids Res.

[4] Casonato S, Cervantes Sanchez A, Haruki H, Rengifo Gonzalez M, Provvedi R, Dainese E, Jaouen T, Gola S, Bini E, Vicente M, Johnsson K, Ghisotti D, Palù G, Hernández-Pando R, Manganelli R (2012). WhiB5, a transcriptional regulator that contributes to Mycobacterium tuberculosis virulence and reactivation. Infect Immun.

[5] Gaudion A, Dawson L, Davis E, Smollett K (2013). Characterisation of the Mycobacterium tuberculosis alternative sigma factor SigG: its operon and regulon. Tuberculosis (Edinb).

[6] Fang H, Yu D, Hong Y, Zhou X, Li C, Sun B (2013). The LuxR family regulator Rv0195 modulates Mycobacterium tuberculosis dormancy and virulence. Tuberculosis (Edinb).

[7] Stewart GR, Snewin VA, Walzl G, Hussell T, Tormay P, O’Gaora P, Goyal M, Betts J, Brown IN, Young DB (2001). Overexpression of heat-shock proteins reduces survival of Mycobacterium tuberculosis in the chronic phase of infection. Nat Med.

[8] Micklinghoff JC, Breitinger KJ, Schmidt M, Geffers R, Eikmanns BJ, Bange FC (2009). Role of the transcriptional regulator RamB (Rv0465c) in the control of the glyoxylate cycle in Mycobacterium tuberculosis. J Bacteriol.

[9] Forrellad MA, Bianco MV, Blanco FC, Nunez J, Klepp LI, Vazquez CL, Santangelo Mde L, Rocha RV, Soria M, Golby P, Gutierrez MG, Bigi F (2013). Study of the in vivo role of Mce2R, the transcriptional regulator of mce2 operon in Mycobacterium tuberculosis. BMC Microbiol.

[10] Cimino M, Thomas C, Namouchi A, Dubrac S, Gicquel B, Gopaul DN (2012). Identification of DNA binding motifs of the Mycobacterium tuberculosis PhoP/PhoR two-component signal transduction system. PLoS One.

[11] Zahrt TC, Song J, Siple J, Deretic V (2001). Mycobacterial FurA is a negative regulator of catalase-peroxidase gene katG. Mol Microbiol.

[12] Gao CH, Yang M, He ZG (2012). Characterization of a novel ArsR-like regulator encoded by Rv2034 in Mycobacterium tuberculosis. PLoS One.

[13] Maciag A, Dainese E, Rodriguez GM, Milano A, Provvedi R, Pasca MR, Smith I, Palu G, Riccardi G, Manganelli R (2007). Global analysis of the Mycobacterium tuberculosis Zur (FurB) regulon. J Bacteriol.

[14] Mendoza Lopez P, Golby P, Wooff E, Nunez Garcia J, Garcia Pelayo MC, Conlon K, Gema Camacho A, Hewinson RG, Polaina J, Suarez Garcia A, Gordon SV (2010). Characterization of the transcriptional regulator Rv3124 of Mycobacterium tuberculosis identifies it as a positive regulator of molybdopterin biosynthesis and defines the functional consequences of a non-synonymous SNP in the Mycobacterium bovis BCG orthologue. Microbiology.

[15] Park HD, Guinn KM, Harrell MI, Liao R, Voskuil MI, Tompa M, Schoolnik GK, Sherman DR (2003). Rv3133c/dosR is a transcription factor that mediates the hypoxic response of *Mycobacterium tuberculosis*. Mol Microbiol.

[16] Kendall SL, Burgess P, Balhana R, Withers M, Ten Bokum A, Lott JS, Gao C, Uhia-Castro I, Stoker NG (2010). Cholesterol utilization in mycobacteria is controlled by two TetR-type transcriptional regulators: kstR and kstR2. Microbiology.

[17] Kendall SL, Withers M, Soffair CN, Moreland NJ, Gurcha S, Sidders B, Frita R, Ten Bokum A, Besra GS, Lott JS, Stoker NG (2007). A highly conserved transcriptional repressor controls a large regulon involved in lipid degradation in Mycobacterium smegmatis and Mycobacterium tuberculosis. Mol Microbiol.

[18] Rickman L, Scott C, Hunt DM, Hutchinson T, Menendez MC, Whalan R, Hinds J, Colston MJ, Green J, Buxton RS (2005). A member of the cAMP receptor protein family of transcription regulators in Mycobacterium tuberculosis is required for virulence in mice and controls transcription of the rpfA gene coding for a resuscitation promoting factor. Mol Microbiol.

[19] Minch KJ, Rustad TR, Peterson E, Winkler J, Brabant W, Hickey M, Reiss D, Ma S, Galagan J, Price N, Baliga NS, Sherman DR: **The gene regulatory network of MTB: context and consequences of protein-DNA interactions.** *Nat Comm* 2014. in press.

[20] Peterson EJR, Reiss DJ, Turkarslan S, Minch KJ, Rustad TR, Sherman DR, Baliga NS (2014). A high resolution network model for global gene regulation in Mycobacterium tuberculosis. Genome Biol.

[21] Lew JM, Kapopoulou A, Jones LM, Cole ST (2011). TubercuList–10 years after. Tuberculosis (Edinb).

[22] **NCBI: Conserved Domains and Protein Classification.** [http://www.ncbi.nlm.nih.gov/Structure/cdd/cdd_help.shtml]

[23] Galagan JE, Minch K, Peterson M, Lyubetskaya A, Azizi E, Sweet L, Gomes A, Rustad T, Dolganov G, Glotova I, Abeel T, Mahwinney C, Kennedy AD, Allard R, Brabant W, Krueger A, Jaini S, Honda B, Yu WH, Hickey MJ, Zucker J, Garay C, Weiner B, Sisk P, Stolte C, Winkler JK, Van de Peer Y, Iazzetti P, Camacho D, Dreyfuss J (2013). The Mycobacterium tuberculosis regulatory network and hypoxia. Nature.

[24] Salgado H, Peralta-Gil M, Gama-Castro S, Santos-Zavaleta A, Muniz-Rascado L, Garcia-Sotelo JS, Weiss V, Solano-Lira H, Martinez-Flores I, Medina-Rivera A, Salgado-Osorio G, Alquicira-Hernández S, Alquicira-Hernández K, López-Fuentes A, Porrón-Sotelo L, Huerta AM, Bonavides-Martínez C, Balderas-Martínez YI, Pannier L, Olvera M, Labastida A, Jiménez-Jacinto V, Vega-Alvarado L, Del Moral-Chávez V, Hernández-Alvarez A, Morett E, Collado-Vides J (2013). RegulonDB v8.0: omics data sets, evolutionary conservation, regulatory phrases, cross-validated gold standards and more. Nucleic Acids Res.

[25] Minch K, Rustad T, Sherman DR (2012). Mycobacterium tuberculosis growth following aerobic expression of the DosR Regulon. PLoS One.

[26] Sherman DR, Voskuil M, Schnappinger D, Liao R, Harrell MI, Schoolnik GK (2001). Regulation of the *Mycobacterium tuberculosis* hypoxic response gene encoding alpha-crystallin. Proc Natl Acad Sci U S A.

[27] Rustad TR, Harrell MI, Liao R, Sherman DR (2008). The enduring hypoxic response of Mycobacterium tuberculosis. PLoS One.

[28] Smoot ME, Ono K, Ruscheinski J, Wang PL, Ideker T (2011). Cytoscape 2.8: new features for data integration and network visualization. Bioinformatics.

[29] Griffin JE, Gawronski JD, Dejesus MA, Ioerger TR, Akerley BJ, Sassetti CM (2011). High-resolution phenotypic profiling defines genes essential for mycobacterial growth and cholesterol catabolism. PLoS Pathog.

[30] Rao SP, Alonso S, Rand L, Dick T, Pethe K (2008). The protonmotive force is required for maintaining ATP homeostasis and viability of hypoxic, nonreplicating Mycobacterium tuberculosis. Proc Natl Acad Sci U S A.

[31] Zhang Y, Heym B, Allen B, Young D, Cole S (1992). The catalase-peroxidase gene and isoniazid resistance of Mycobacterium tuberculosis. Nature.

[32] Pym AS, Domenech P, Honore N, Song J, Deretic V, Cole ST (2001). Regulation of catalase-peroxidase (KatG) expression, isoniazid sensitivity and virulence by furA of Mycobacterium tuberculosis. Mol Microbiol.

[33] Beste DJ, Hooper T, Stewart G, Bonde B, Avignone-Rossa C, Bushell ME, Wheeler P, Klamt S, Kierzek AM, McFadden J (2007). GSMN-TB: a web-based genome-scale network model of Mycobacterium tuberculosis metabolism. Genome Biol.

[34] Beste DJ, Bonde B, Hawkins N, Ward JL, Beale MH, Noack S, Noh K, Kruger NJ, Ratcliffe RG, McFadden J (2011). (13)C metabolic flux analysis identifies an unusual route for pyruvate dissimilation in mycobacteria which requires isocitrate lyase and carbon dioxide fixation. PLoS Pathog.

[35] **BEI Resources Strain Depository.** [www.beiresources.org]

[36] **Dynamic Excel spreadsheet for querying TFOE and ChIP-seq data from this study.** [http://networks.systemsbiology.net/mtb]

[37] Ehrt S, Guo XV, Hickey CM, Ryou M, Monteleone M, Riley LW, Schnappinger D (2005). Controlling gene expression in mycobacteria with anhydrotetracycline and Tet repressor. Nucleic Acids Res.

[38] Rustad T, Roberts D, Liao R, Sherman DR, Parish T, Brown A (2007). RNA Isolation. Mycobacteria Protocols Handbook.

[39] Bolstad BM, Irizarry RA, Astrand M, Speed TP (2003). A comparison of normalization methods for high density oligonucleotide array data based on variance and bias. Bioinformatics.

[40] Schellenberger J, Que R, Fleming RM, Thiele I, Orth JD, Feist AM, Zielinski DC, Bordbar A, Lewis NE, Rahmanian S, Kang J, Hyduke DR, Palsson BØ (2011). Quantitative prediction of cellular metabolism with constraint-based models: the COBRA Toolbox v2.0. Nat Protoc.

[41] Shlomi T, Cabili MN, Herrgard MJ, Palsson BO, Ruppin E (2008). Network-based prediction of human tissue-specific metabolism. Nat Biotechnol.

[42] Zur H, Ruppin E, Shlomi T (2010). iMAT: an integrative metabolic analysis tool. Bioinformatics.

