# Supplementary figures and images for: Mapping and manipulating the Mycobacterium tuberculosis transcriptome using a transcription factor overexpression-derived regulatory network
Source: Genome Biol. 2014 Nov 3;15(11):502. doi: 10.1186/s13059-014-0502-3 (PMC4249609; doi:10.1186/s13059-014-0502-3)

## Slide 1
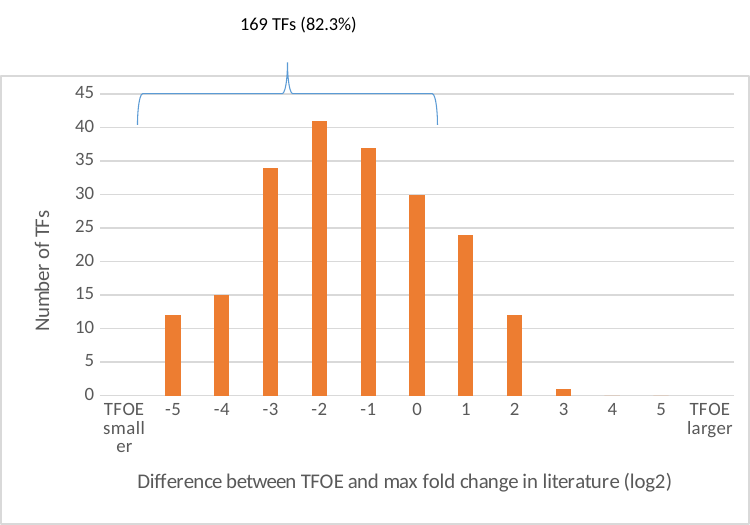

169 TFs (82.3%)
### Chart
| Category | |
|---|---|
| TFOE smaller | None |
| -5 | 12.0 |
| -4 | 15.0 |
| -3 | 34.0 |
| -2 | 41.0 |
| -1 | 37.0 |
| 0 | 30.0 |
| 1 | 24.0 |
| 2 | 12.0 |
| 3 | 1.0 |
| 4 | 0.0 |
| 5 | 0.0 |
| TFOE larger | None |

Supplement: Additional file 2: Figure S1. — Comparison of TFOE induction to previously published expression analyses. For each TF the largest fold change was found among the collection of 2,483 expression profiles and that change was compared to the TFOE induction. A histogram of the differences shows a large majority of TFs are not induced beyond what is seen in at least one other condition (the bins under the bracket). [file 13059_2014_502_MOESM2_ESM.pptx]
